# Capacitance-Based Characterization of Air-Void Distribution in Asphalt Mixtures Using a Saturated Reference Field

**DOI:** 10.3390/s26154961

**Published:** 2026-08-05

**Authors:** Xing Hu, Qiao Dong, Bin Shi, Kang Yao, Zhen Liu

**Affiliations:** 1 Department of Road Engineering, Southeast University, Nanjing 211189, China; huxing1108@163.com (X.H.); yaokang0714@163.com (K.Y.); 2 National Demonstration Center for Experimental Road and Traffic Engineering Education, Nanjing 211189, China; 3 Department of Civil Engineering, Hefei University of Technology, Hefei 230009, China; binshi@hfut.edu.cn; 4 Institute of Space and Earth Information Science, The Chinese University of Hong Kong, Hong Kong SAR, China

**Keywords:** asphalt mixture, air-void distribution, saturated reference field, capacitance measurement, annular capacitive sensor

## Abstract

Air-void distribution is an important internal characteristic of asphalt mixtures, as it affects compaction quality, moisture susceptibility, permeability, and long-term pavement durability. Conventional air-void testing methods generally provide only an average volumetric parameter and cannot effectively describe the spatial distribution of air voids within cylindrical specimens. To address this limitation, this study proposes a capacitance-based method for characterizing the vertical and radial air-void distribution of asphalt mixtures using a saturated reference field. An annular capacitive sensor was designed for cylindrical asphalt mixture specimens, and its structural dimensions were optimized using capacitance sensitivity and sensitivity-field distribution uniformity as evaluation indicators. Asphalt mixture specimens with different gradations and compaction conditions were prepared and tested under a saturated reference-field measurement scheme. Dielectric indicators derived from capacitance measurements were used to characterize the variation in air-void distribution along the specimen height and across radial regions. Layer-wise air-void measurements were further conducted to validate the vertical distribution results, while radial partition-based indicators were introduced to quantitatively describe the air-void distribution characteristics from the center to the edge of the specimen. In addition, rotation-angle and saturated-condition stability tests were performed to evaluate the robustness of the proposed method. The results indicate that the saturated reference-field capacitance method can effectively reflect the spatial variation in air voids in asphalt mixtures and provides a low-cost, rapid, and non-destructive approach for evaluating air-void distribution characteristics in laboratory-compacted specimens.

## 1. Introduction

Air voids are one of the most important internal structural characteristics of asphalt mixtures. Their content, connectivity, and spatial distribution directly affect compaction quality, permeability, moisture susceptibility, load transfer, and long-term pavement durability [1,2,3,4]. In practice, the air-void content of asphalt mixtures is usually controlled as an average volumetric parameter [5]. However, asphalt mixture specimens are inherently heterogeneous [6]. Even when two specimens have similar bulk air-void contents, their internal void distributions may be substantially different. Localized void concentration may accelerate water intrusion, stripping, raveling, and fatigue damage [7,8]. Therefore, characterizing the spatial distribution of air voids, rather than only measuring the overall air-void content, is important for evaluating mixture structure and compaction quality [9].

Conventional air-void measurements, such as the saturated surface-dry method and the volumetric method, are widely used because of their simplicity and standardization [10,11,12]. These methods provide reliable bulk air-void content for dense-graded and open-graded mixtures. Nevertheless, they cannot describe the variation in air voids along the specimen height or across the radial direction [13]. Layer cutting can provide limited information on vertical air-void distribution, but it is destructive and cannot be used for repeated measurement of the same specimen [14]. X-ray computed tomography has been used to reconstruct the three-dimensional void structure of asphalt mixtures and can provide detailed information on void morphology, connectivity, and distribution [15,16]. However, CT scanning is expensive, time-consuming, and not always convenient for routine laboratory testing or rapid compaction evaluation [17,18]. These limitations motivate the development of a low-cost, rapid, and non-destructive method for characterizing air-void distribution in asphalt mixtures [19,20].

Electrical and capacitive sensing methods provide a promising alternative for non-destructive characterization of heterogeneous materials [21]. The dielectric response of asphalt mixtures is affected by aggregates, asphalt mortar, air voids, and moisture [22,23]. When the internal void structure changes, the effective dielectric distribution also changes [24,25]. Electrical capacitance tomography and capacitive sensing can, therefore, be used to infer the spatial variation in material properties from boundary capacitance measurements [26]. Compared with CT, capacitive sensing has simpler hardware, lower cost, and faster data acquisition [27,28]. It is also suitable for repeated measurements at different heights [29]. In representative ECT studies of gas–liquid flows, the higher-permittivity liquid generally forms the continuous or background phase, while the lower-permittivity gas occupies mobile dispersed or segregated regions. The reconstructed dielectric field, therefore, describes a time-varying fluid-phase distribution [30,31]. A compacted asphalt mixture has a distinctly different dielectric phase structure. Mineral aggregates and asphalt mortar form a rigid and spatially heterogeneous solid skeleton, whereas air voids are fixed within this skeleton and may form connected, partially connected, or isolated pore structures [32,33]. Their size, connectivity, and spatial distribution are governed by aggregate gradation, compaction method, and compaction effort [34]. Consequently, the measured capacitance reflects the coupled dielectric response of a heterogeneous solid skeleton and a stationary pore network rather than the instantaneous distribution of two mobile fluid phases. However, applying capacitance-based methods to asphalt mixtures remains challenging because the dielectric contrast between dry air voids and the asphalt–aggregate skeleton is limited. In addition, an asphalt mixture is a heterogeneous composite, and its fully dense reference state cannot be directly measured. As a result, conventional air-field reconstruction often requires a simulated full-field matrix, which may introduce additional reconstruction errors.

To overcome these limitations, this study proposes a saturated-field capacitance measurement method for air-void distribution characterization. The basic idea is to use water as a high-permittivity filling medium for connected air voids. Water preferentially enters accessible connected pores, whereas isolated or poorly connected pores may remain partly air-filled. After saturation, the resulting water-filled regions produce a much stronger dielectric response than dry air voids. This increases the contrast between void-rich and void-poor regions and improves the sensitivity of capacitance measurements to air-void structures. More importantly, in the saturated-field method, both the empty-field and full-field capacitance matrices can be obtained experimentally: the empty field is measured in air, and the full field is measured after the acrylic cylinder is filled with water. This avoids the need to approximate the full-field matrix using a homogeneous asphalt mixture model. Therefore, the saturated-field method provides a more physically defined reference system for dielectric reconstruction.

A movable annular capacitive sensor was designed in this study to enable depth-wise scanning of cylindrical asphalt mixture specimens. The sensor consists of an open-top acrylic cylinder, a flexible substrate, and twelve measuring electrodes. Unlike fixed multi-layer electrode systems, the movable annular structure can collect capacitance data at different height positions while reducing the structural complexity and potential interlayer coupling. Finite-element simulation was used to optimize the sensor configuration by considering capacitance sensitivity and sensitivity-field distribution. The optimized sensor was then used to characterize asphalt mixtures with different gradations and compaction states. The combination of the movable electrode layer and the saturated reference field enables the dielectric response to be examined at multiple cross-sections and related to compaction-induced variations in both the vertical and radial directions.

The main objectives of this study are as follows:

(1) To develop a movable annular capacitive sensor and optimize its structural parameters using a finite-element simulation;

(2) To establish a saturated-field capacitance measurement procedure for asphalt mixture specimens;

(3) To characterize the vertical air-void distribution using the capacitance sum and mean meso-scale dielectric constant;

(4) To quantify radial air-void distribution using the edge-to-center ratio and radial dielectric gradient; 

(5) To validate the proposed method using layer-wise air-void measurements and repeatability tests.

## 2. Materials and Methods

### 2.1. Specimen Preparation

#### 2.1.1. Materials and Gradation Design

Basalt aggregates were supplied by Jiangsu Judeng Construction Engineering Co., Ltd. (Zhenjiang, Jiangsu, China), and limestone mineral filler was obtained from Nanjing China United Cement Co., Ltd. (Nanjing, Jiangsu, China). Two asphalt binders were selected according to the mixture type. SBS-modified asphalt supplied by Shandong Chambroad Petrochemicals Co., Ltd. (Binzhou, Shandong, China) was used for AC-13 and SMA-13, whereas high-viscosity asphalt from the same manufacturer was used for PAC-13 and PAC-20. The basic properties of the two binders are listed in Table 1.

**Figure 1:**
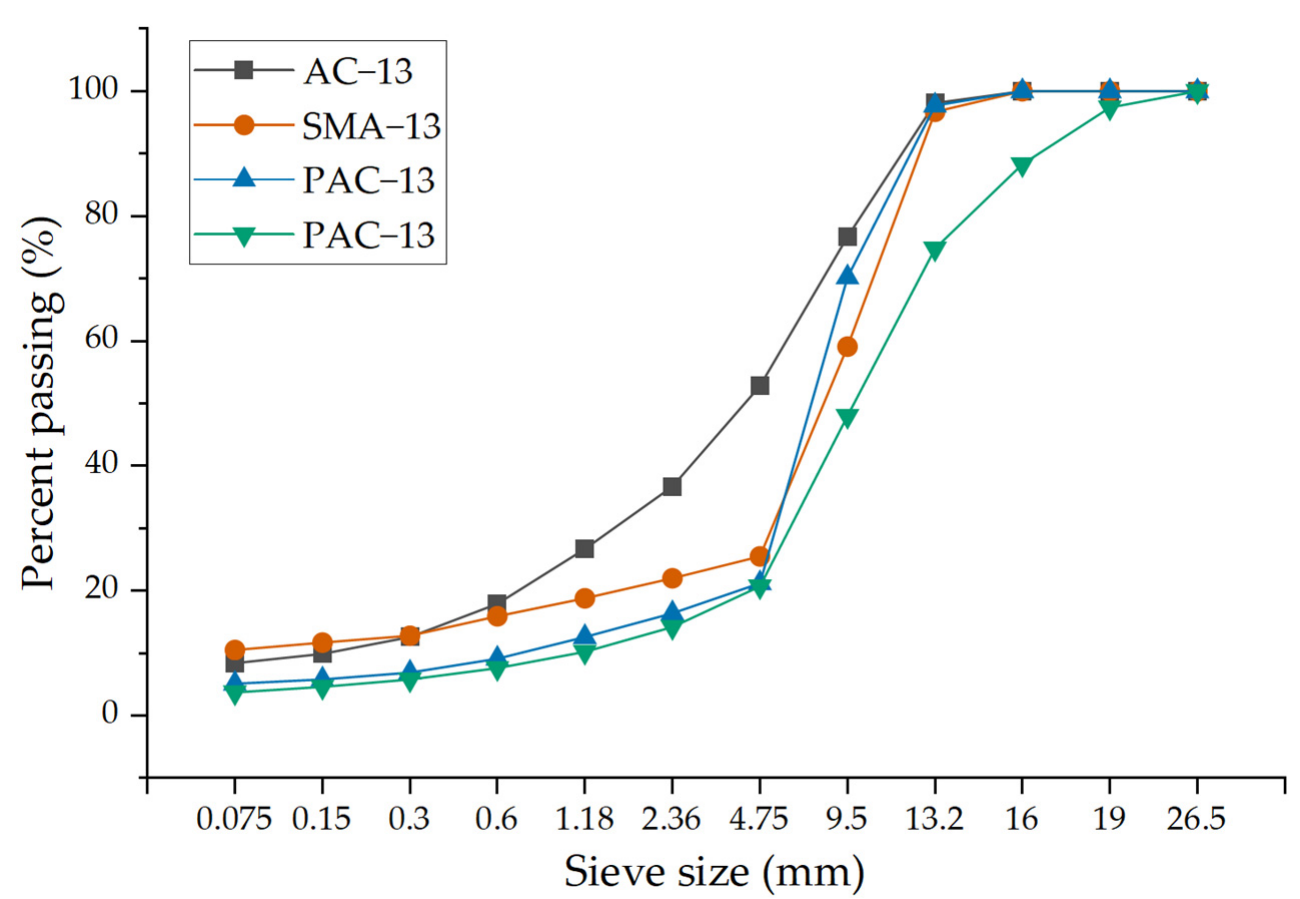
Gradation curves of AC-13, SMA-13, PAC-13, and PAC-20 asphalt mixtures.

Four mixtures were designed, as follows: AC-13, SMA-13, PAC-13, and PAC-20. AC-13 was used as a dense-graded reference mixture. SMA-13 represented a stone mastic asphalt mixture with a skeleton-dense structure. PAC-13 and PAC-20 were selected to provide porous mixtures with higher and more connected air-void contents. The gradation curves are shown in Figure 1. The selected gradations cover dense-graded, skeleton-dense, and open-graded structures, providing specimens with different void contents and void connectivity for capacitance-based characterization.

#### 2.1.2. Compaction Methods and Specimen Groups

Two specimen series were prepared. The first series included AC-13, SMA-13, and PAC-13 specimens compacted by the Marshall compaction method. This series was used to compare air-void distribution characteristics among mixtures with different gradations. The second series consisted of PAC-20 specimens prepared by gyratory compaction. Three compaction levels were adopted, with 40, 60, and 80 gyrations. This series was used to examine the influence of compaction level on air-void distribution.

Three independently prepared specimens were tested for each mixture type or compaction level. For the Marshall-compacted series, three AC-13, three SMA-13, and three PAC-13 specimens were prepared. For the gyratory-compacted PAC-20 series, three specimens were prepared at each of the 40-, 60-, and 80-gyration levels. Therefore, a total of 18 specimens were included in the capacitance measurements. Each specimen was tested using both the air-field and saturated-field methods. Capacitance data were collected at six cross-sections located at 5, 15, 25, 35, 45, and 55 mm above the specimen bottom. The results for each mixture type or compaction level were obtained by averaging the corresponding measurements from the three independently prepared specimens. After compaction and cooling, the height of each cylindrical specimen were measured at multiple positions using a vernier caliper. The average values were recorded as the specimen dimensions. The detailed specimen information is summarized in Table 2.

### 2.2. Measurement System and Principle

#### 2.2.1. Annular Capacitive Sensor

Air-void distribution in asphalt mixture specimens can vary along the height direction. Therefore, the sensor should be able to cover the specimen depth during measurement. Instead of using a multi-layer electrode configuration, which may increase interlayer coupling, a movable annular capacitive sensor was designed for air-void characterization.

**Figure 2:**
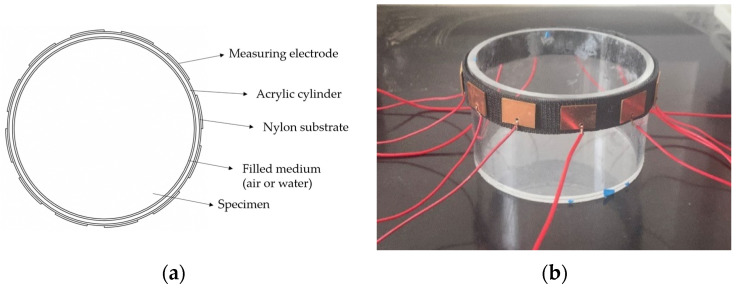
Movable annular capacitive sensor: (**a**) schematic diagram; (**b**) photograph.

The sensor consisted of an open-top acrylic cylinder, twelve measuring electrodes, and a flexible nylon substrate. The acrylic cylinder served as the internal support and water-containing chamber for the saturated reference-field measurement. The measuring electrodes were attached to the flexible nylon substrate using conductive adhesive. During testing, the flexible electrode layer moved along the outer surface of the acrylic cylinder, allowing capacitance data to be collected at different height positions. This design enabled depth-wise scanning while reducing the structural complexity associated with fixed multi-layer electrodes. The schematic diagram and a photograph of the sensor are shown in Figure 2.

The diameters of both the Marshall-compacted and gyratory-compacted specimens were approximately 100 mm. Because the inner diameter of the acrylic cylinder was slightly larger than the specimen’s diameter, a small annular gap between the specimen and the cylinder wall was unavoidable. The same cylinder was used throughout the experiments. After specimen placement, the radial clearances were measured at four circumferential positions separated by approximately 90° using a tapered gap gauge. The specimen position was adjusted until the four measured clearances were approximately equal, thereby maintaining a nearly concentric configuration.

#### 2.2.2. Measurement Principle and Finite-Element Simulation

**Figure 3:**
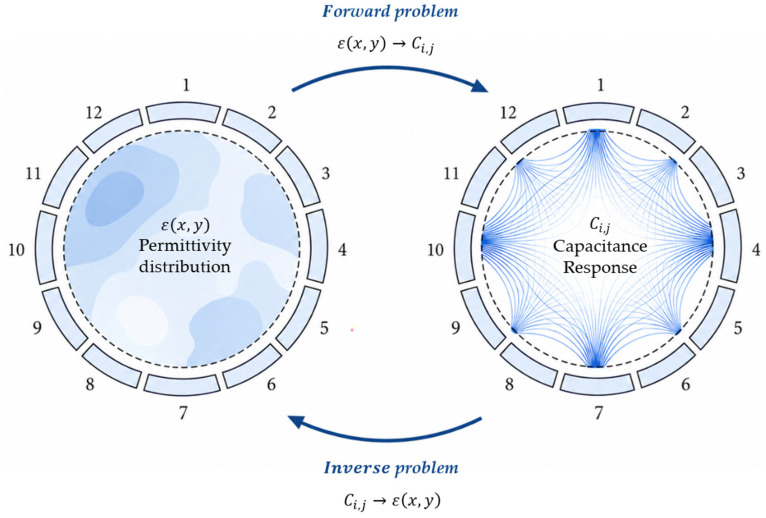
Schematic diagram of the forward and inverse problems.

The capacitance-based characterization process can be described as a forward and inverse problem, as shown in Figure 3. In the forward problem, the sensor structure, material distribution, and electrode excitation scheme are defined to calculate the electric field, potential distribution, capacitance matrix, and sensitivity field. In the inverse problem, the measured capacitance data are combined with the sensitivity field to estimate the dielectric distribution inside the sensing domain. Since the dielectric distribution is affected by aggregates, asphalt mortar, air voids, and water-filled voids under saturated conditions, the reconstructed dielectric response can be used to characterize the air-void distribution of asphalt mixtures.

For a given electrode pair, the capacitance is determined by the permittivity distribution and potential field in the sensing domain, as follows:

(1) $$C_{i,j}=\frac{Q}{V}=\frac{1}{V}\iint_{\Gamma }\varepsilon \left(x,y\right)\nabla \Phi \left(x,y\right)d\Gamma$$

where $C_{i,j}$ is the capacitance between electrodes i and j, Q is the induced charge, V is the potential difference between the electrode pair, $\varepsilon \left(x,y\right)$ is the permittivity distribution, $\Phi \left(x,y\right)$ is the potential distribution, and Γ denotes the electrode surface.

The measured capacitance can be further expressed as an integral form related to the sensitivity field, as follows:

(2) $$C=\iint_{\Gamma }\varepsilon \left(x,y\right)\cdot S\left(x,y,\varepsilon \left(x,y\right)\right)dxdy$$

where $S\left(x,y,\varepsilon \left(x,y\right)\right)$ is the sensitivity field. The sensitivity field reflects the contribution of local dielectric variation to the measured capacitance. Regions with higher sensitivity have a stronger influence on the capacitance response when their permittivity changes.

In this study, the sensitivity field was obtained using finite-element simulation. For electrode pair i−j, the sensitivity field can be calculated from the electric field distributions generated by the following two electrodes:

(3) $$S_{i,j}\left(x,y\right)=-\int_{p\left(x,y\right)}\frac{E_{i}\left(x,y\right)}{V_{i}}\cdot \frac{E_{j}\left(x,y\right)}{V_{j}}dxdy$$

where $S_{i,j}\left(x,y\right)$ is the sensitivity value at position $\left(x,y\right)$, $E_{i}\left(x,y\right)$ and $E_{j}\left(x,y\right)$ are the electric field intensities when electrodes i and j are excited, respectively, and $V_{i}$ and $V_{j}$ are the corresponding excitation voltages. This formulation links the capacitance response to the local electric field distribution and provides a basis for evaluating sensor performance.

A finite-element electrostatic model was therefore established using COMSOL Multiphysics^®^ version 6.4 to calculate the capacitance response and sensitivity-field distribution of the annular capacitive sensor. The model included the asphalt mixture specimen, open-top acrylic cylinder, flexible substrate, and measuring electrodes. One electrode was excited in each calculation, while the remaining electrodes were grounded. The excitation electrode was sequentially switched to obtain the capacitance matrix and sensitivity-field distribution of different electrode pairs.

The following two indicators were used to evaluate the sensor structure: capacitance sensitivity and sensitivity-field distribution uniformity. Capacitance sensitivity was used to quantify the response of the sensor to dielectric variation and was calculated as follows:

(4) $$S_{C}=\frac{\left|C_{h}-C_{l}\right|}{C_{l}}$$

where $S_{C}$ is the capacitance sensitivity, $C_{h}$ is the capacitance under the high-permittivity reference state, and $C_{l}$ is the capacitance under the low-permittivity reference state.

Sensitivity-field distribution uniformity was used to evaluate the spatial balance of the sensing field in the measured cross-section. It was defined as follows:

(5) $$\left\{\begin{array}{c} S_{i,j}^{avg}=\frac{1}{n}\sum_{e=1}^{n}S_{i,j}\left(k\right)\\ S_{i,j}^{dev}={\left\{\frac{1}{n-1}\sum_{e=1}^{n}{\left[S_{i,j}\left(k\right)-S_{i,j}^{avg}\right]}^{2}\right\}}^{1/2}\\ D_{s}=\frac{2}{P}\left(\sum_{i=1}\sum_{j=2}^{\frac{2}{P}+1}\left|\frac{S_{i,j}^{dev}}{S_{i,j}^{avg}}\right|\right) \end{array}\right.$$

where $S_{i,j}^{avg}$ is the average sensitivity; $S_{i,j}^{dev}$ is the standard deviation of n micro-elements in the measured area; $D_{s}$ is defined as the sensitivity distribution coefficient; and P is the number of the electrode plates. A smaller value of $D_{s}$ indicates a more uniform distribution of sensitivity.

Based on the finite-element model, structural parameters of the annular capacitive sensor, including electrode thickness, substrate thickness, and electrode width, were varied to analyze their effects on capacitance sensitivity and sensitivity-field uniformity.

#### 2.2.3. Capacitance Acquisition System

**Figure 4:**
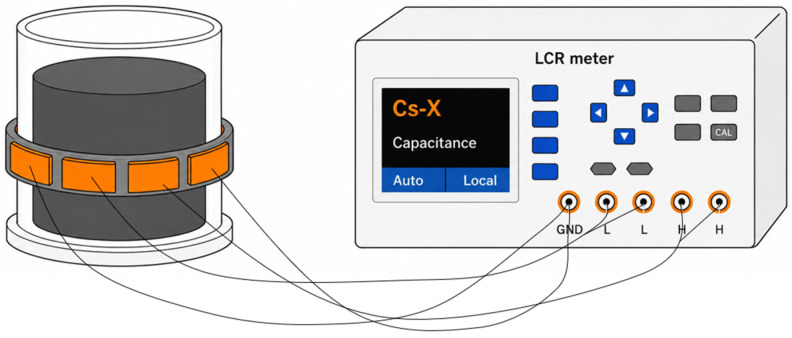
Schematic diagram of the capacitance measurement system.

Capacitance measurements were performed using an LCR meter (VICTOR 4090A, henzhen Victor Hi-Tech Co., Ltd., Shenzhen, China). For the twelve-electrode sensor, 66 independent electrode-pair capacitances were measured sequentially to form one capacitance dataset at each height position. The test frequency and excitation voltage were set as 100 kHz and 2 V, respectively. The schematic diagram of the capacitance measurement system is shown in Figure 4.

Before testing, the LCR meter was calibrated to reduce the influence of stray capacitance from cables and connectors. Each capacitance value was measured repeatedly, and the average value was used for subsequent analysis. All measurements were conducted using the same LCR meter, cables, excitation frequency, and excitation voltage under consistent laboratory conditions. The electrode layer was fixed at each prescribed height before capacitance acquisition to reduce variations associated with electrode positioning.

### 2.3. Saturated Measurement and Evaluation Procedure

#### 2.3.1. Saturated Reference-Field Measurement

**Figure 5:**
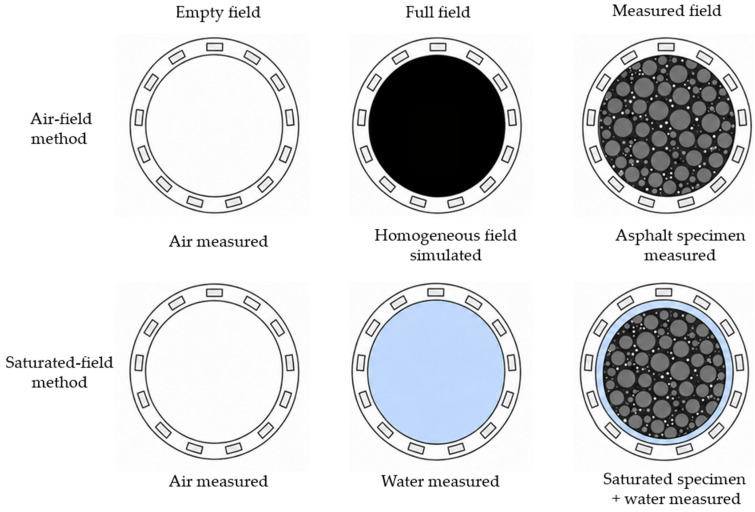
Capacitance measurement configurations for the air-field and saturated-field methods.

The following two measurement methods were compared in this study: the air-field method and the saturated-field method. The measurement configurations for the air-field and saturated-field methods are shown in Figure 5. In the air-field method, the empty field was defined as the air-filled sensing region without a specimen, and its capacitance matrix was measured experimentally. The measured field was obtained after placing the asphalt mixture specimen inside the sensor. However, the full-field matrix of the air-field method could not be directly measured, because an asphalt mixture is a heterogeneous composite and its internal air voids cannot be completely removed. Therefore, the full field was approximated by numerical simulation. In the simulation, the sensing region was assumed to be homogeneous, with a relative permittivity of 9. This value was selected according to the measured permittivity range of asphalt mixtures, generally between 5 and 9. This approximation may introduce reconstruction errors.

Previous studies have shown that capacitive sensors are sensitive to moisture variation. Based on this characteristic, a saturated-field method was proposed for air-void characterization. In this method, the empty-field matrix was measured when the sensing region was filled with air and no specimen was placed inside the sensor. The full-field matrix was measured after the acrylic cylinder was filled with water to its upper surface. For the measured field, the asphalt mixture specimens were first fully immersed in water for 24 h to facilitate water penetration into the accessible connected voids. During the final stage of immersion, each specimen was periodically removed from the water, gently wiped with a damp cloth to remove visible surface water, and weighed. Saturation was considered to have been achieved when the mass change between two consecutive measurements was less than 0.1%. The saturated specimen was then immediately transferred into the acrylic cylinder, and water was added until it reached the upper surface of the cylinder. After standing for 3–5 min, the capacitance matrix of the saturated specimen with surrounding water was recorded. Visible air bubbles trapped between the specimen surface and the cylinder wall were removed before data acquisition. The concentric position of the specimen was maintained throughout the height-wise scanning procedure. Because water penetration depends on pore accessibility and connectivity, the saturated-field response primarily enhances the contribution of water-accessible connected voids, while isolated or poorly connected voids may remain partly air-filled.

For both methods, the movable annular electrode layer was positioned at selected height locations along the specimen. At each height, all independent electrode-pair capacitances were collected to form one capacitance matrix. The electrode layer was then moved along the height direction to obtain capacitance responses at different depths. The same scanning procedure was used for the air-field and saturated-field methods to ensure comparability.

#### 2.3.2. Indicators for Air-Void Distribution Characterization

The measured capacitance matrix was first converted into a dimensionless form using Maxwell normalization. This treatment reduces the influence of sensor geometry, boundary conditions, and reference-field differences. For electrode pair i−j, the Maxwell-normalized capacitance was calculated as follows:

(6) $$C_{M,ij}=\frac{C_{ij}^{m}-C_{ij}^{e}}{C_{ij}^{f}-C_{ij}^{e}}\frac{C_{ij}^{f}+2C_{ij}^{e}}{C_{ij}^{m}+2C_{ij}^{e}}$$

where $C_{M,ij}$ is the Maxwell-normalized capacitance measurement. $C_{ij}^{m}$, $C_{ij}^{e}$ and $C_{ij}^{f}$ are the measured-field, empty-field, and full-field capacitance measurements, respectively.

For the vertical air-void characterization, the following two indicators were used: the capacitance sum, $C_{s}$, and the mean meso-scale dielectric constant, $\varepsilon_{m}$. At a given height, z, Cs was calculated from all independent electrode pairs, as follows:

(7) $$C_{s}\left(z\right)=\sum_{i<j}C_{M,ij}\left(z\right)$$

where $C_{M,ij}\left(z\right)$ is the Maxwell-normalized capacitance at height z. The capacitance sum represents the overall dielectric response of the measured cross-section.

The mean meso-scale dielectric constant was calculated from the reconstructed dielectric field, as follows:

(8) $$\varepsilon_{m}\left(z\right)=\frac{1}{N}\sum_{p=1}^{N}\varepsilon_{p}\left(z\right)$$

**Figure 6:**
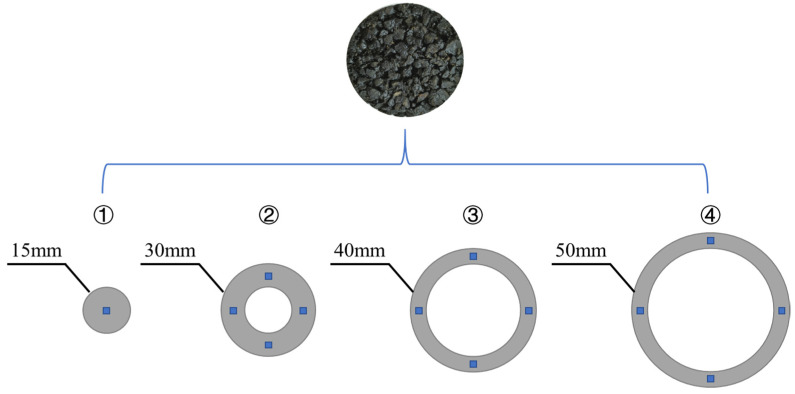
Radial partitioning of the reconstructed dielectric field.

For radial characterization, the reconstructed dielectric field was divided into four radial regions, as shown in Figure 6. The representative radii were 15, 30, 40, and 50 mm. For the central region, the average value of all pixels within the radius of 15 mm was used, as follows:

(9) $$\varepsilon_{r,1}=\frac{1}{N_{1}}\sum_{p\in R_{1}}\varepsilon_{p}$$

where $\varepsilon_{r,1}$ is the characteristic dielectric constant of the central region, $R_{1}$ is the central region, and $N_{1}$ is the number of pixels in this region.

For the other three regions, four representative pixels were selected along the upper, lower, left, and right directions. Their average value was used as the characteristic dielectric constant of each radial region, as follows:

(10) $$\varepsilon_{r,k}=\frac{1}{4}\left(\varepsilon_{k}^{up}+\varepsilon_{k}^{down}+\varepsilon_{k}^{left}+\varepsilon_{k}^{right}\right),k=2,3,4$$

where $\varepsilon_{r,k}$ is the characteristic dielectric constant of the k-th radial region. The radial sequence $\varepsilon_{r,1}$, $\varepsilon_{r,2}$, $\varepsilon_{r,3}$ and $\varepsilon_{r,4}$ was used to describe the air-void distribution from the center to the edge of the specimen.

To further quantify the radial variation, the edge-to-center ratio and radial dielectric gradient were defined. The edge-to-center ratio and the radial dielectric gradient were calculated as follows:

(11) $$R_{e/c}=\frac{\varepsilon_{r,4}}{\varepsilon_{r,1}}$$

(12) $$G_{r}=\frac{\varepsilon_{r,4}-\varepsilon_{r,1}}{r_{4}-r_{1}}$$

where $R_{e/c}$ is the edge-to-center ratio, $G_{r}$ is the radial dielectric gradient, and $r_{1}$ and $r_{4}$ are the representative radii of the central and edge regions, respectively. $R_{e/c}$ describes the relative dielectric level between the specimen edge and center. A larger absolute value of $G_{r}$ indicates a stronger radial variation in the reconstructed dielectric field. These two indicators were used to compare the radial air-void distribution characteristics among different specimens.

#### 2.3.3. Validation and Repeatability Tests

To validate the vertical characterization results at the layer scale, the same specimens were first measured using the capacitance-based method and were subsequently cut into lower, middle, and upper layers. The air-void content of each layer was then measured and compared with the capacitance-based indicators obtained from the corresponding height range. This one-to-one correspondence between the capacitance measurements and the destructive reference measurements avoided the uncertainty caused by specimen-to-specimen variability. For the AC-13 and SMA-13 mixtures, the layer-wise air-void contents were determined using the saturated surface-dry method. For the PAC-13 and PAC-20 mixtures, the volumetric method was used because of their open-graded structures and high connected air-void contents.

Repeatability tests were conducted to evaluate the stability of the capacitance measurement. The following two factors were considered: specimen rotation angle and saturated condition.

For the rotation-angle test, the same saturated specimen was placed in the annular capacitive sensor at a fixed height. The specimen was then rotated to 0°, 90°, 180°, and 270°. At each angle, a complete capacitance matrix was recorded. This test was used to examine the influence of specimen orientation on the measured capacitance response.

For the saturated-condition test, the saturated specimen was placed inside the acrylic cylinder, and water was added to the upper surface of the cylinder. After a short standing period, repeated measurements were performed at the same height. The test was used to assess the stability of the saturated reference field and the influence of water redistribution during measurement.

The repeatability was evaluated using the coefficient of variation and relative deviation. For a given indicator, X, such as $C_{s}$, $\varepsilon_{m}$, $R_{e/c}$, or $G_{r}$, the coefficient of variation was calculated as follows:

(13) $$CV=\frac{\sigma_{X}}{\bar{X}}\times 100\%$$

where $\bar{X}$ is the mean value of repeated measurements, and $\sigma_{X}$ is the corresponding standard deviation.

The relative deviation of each measurement was calculated as follows:

(14) $$RD_{i}=\frac{\left|X_{i}-\bar{X}\right|}{\bar{X}}\times 100\%$$

where $X_{i}$ is the value obtained from the i-th measurement. Lower values of CV and $RD_{i}$ indicate better measurement repeatability.

## 3. Results and Discussion

### 3.1. Optimization of Sensor Structure Based on Finite-Element Simulation

#### 3.1.1. Influence of Structural Parameters on Sensor Performance

The effects of electrode width, substrate thickness, and electrode thickness on sensor performance were evaluated using finite-element simulation. The following two indicators were used: capacitance sensitivity, $S_{c}$, and sensitivity distribution coefficient, $D_{s}$. A higher $S_{c}$ indicates a stronger capacitance response to dielectric variation, whereas a lower $D_{s}$ indicates a more uniform sensitivity-field distribution in the sensing region.

The ranges of the structural parameters were determined according to the practical fabrication and installation constraints of the sensor. The upper limit of the electrode width was constrained by the spacing between adjacent electrodes and the available bonding area. The lower limits of the substrate thickness and electrode thickness were determined by the material strength, fabrication accuracy, and bonding stability. Within the allowable range of each parameter, several evenly spaced levels were selected for the single-factor analysis. In each simulation set, only one structural parameter was varied, while the other parameters were fixed at the middle levels of their corresponding ranges. This setting was used to reduce parameter coupling and clarify the independent effect of each structural parameter on the sensor performance.

**Figure 7:**
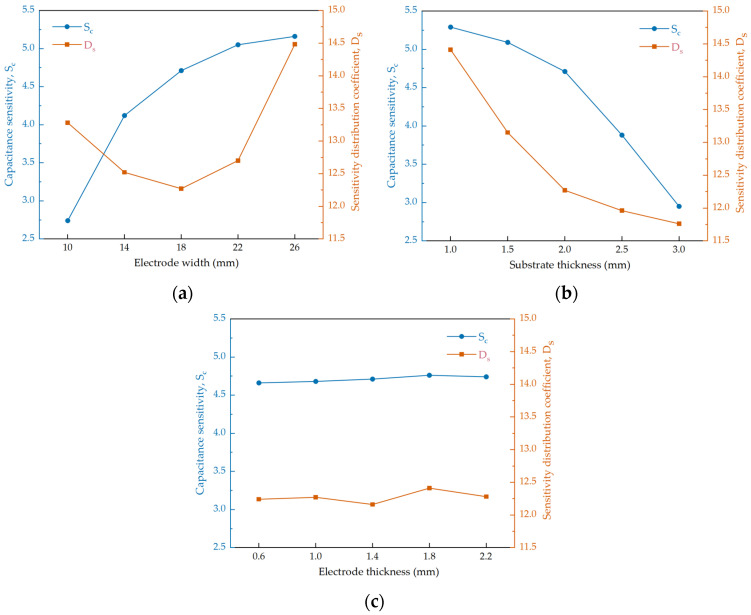
Effects of structural parameters on capacitance sensitivity and sensitivity distribution coefficient: (**a**) electrode width; (**b**) substrate thickness; (**c**) electrode thickness.

As shown in Figure 7a, $S_{c}$ increased with the electrode width. When the electrode width increased from 10 mm to 26 mm, $S_{c}$ increased from 2.74 to 5.16. This indicates that a larger electrode width enhanced the electric-field coupling between the electrodes and the sensing region. However, $D_{s}$ first decreased and then increased. The minimum $D_{s}$ appeared at an electrode width of 18 mm. When the electrode width further increased to 26 mm, $D_{s}$ increased to 14.48. This suggests that an excessively large electrode width caused the sensitivity field to become more concentrated near the electrodes. Therefore, electrode width should not be selected only by maximizing $S_{c}$. Figure 7b shows the influence of substrate thickness. With an increasing substrate thickness, $S_{c}$ decreased from 5.29 to 2.95. This is because a thicker substrate increased the distance between the electrodes and the sensing region, thereby weakening electric-field coupling. In contrast, $D_{s}$ decreased from 14.41 to 11.76, indicating that a thicker substrate produced a more uniform sensitivity-field distribution. However, this improvement in field uniformity was achieved at the cost of reduced capacitance sensitivity. Thus, substrate thickness should be determined by balancing signal response and field uniformity. As shown in Figure 7c, electrode thickness had a limited influence on sensor performance. $S_{c}$ changed only slightly within the tested range, and $D_{s}$ also showed minor fluctuations. This indicates that once sufficient electrical conductivity was ensured, further increasing electrode thickness did not substantially improve the capacitance response or the sensitivity-field distribution. Therefore, electrode thickness was mainly governed by fabrication feasibility, flexibility, and bonding stability.

Overall, the three structural parameters affected the sensor performance in different ways. Electrode width mainly controlled capacitance response and local field concentration. Substrate thickness mainly affected the coupling distance between the electrodes and the sensing region. Electrode thickness had a relatively weak effect. Since a higher $S_{c}$ may be accompanied by a higher $D_{s}$, the optimized configuration cannot be determined from a single indicator.

#### 3.1.2. Determination of the Optimized Sensor Configuration

To determine the optimized sensor configuration, the original indicators were converted into normalized scores. Capacitance sensitivity, $S_{c}$, was treated as a positive indicator, while sensitivity-field non-uniformity, $D_{s}$, was treated as a negative indicator. Therefore, $S_{c}$ was normalized in the forward direction, and $D_{s}$ was normalized in the reverse direction, as follows:

(15) $$S_{C}^{*}=\frac{S_{C}-S_{C,min}}{S_{C,max}-S_{C,min}}$$

(16) $$D_{S}^{*}=\frac{D_{S,max}-D_{S}}{D_{S,max}-D_{S,min}}$$

where $S_{C}^{*}$ is the normalized capacitance sensitivity score, and $D_{S}^{*}$ is the normalized sensitivity-field uniformity score. After normalization, both indicators were converted into benefit-type indices, with larger values indicating better performance.

**Figure 8:**
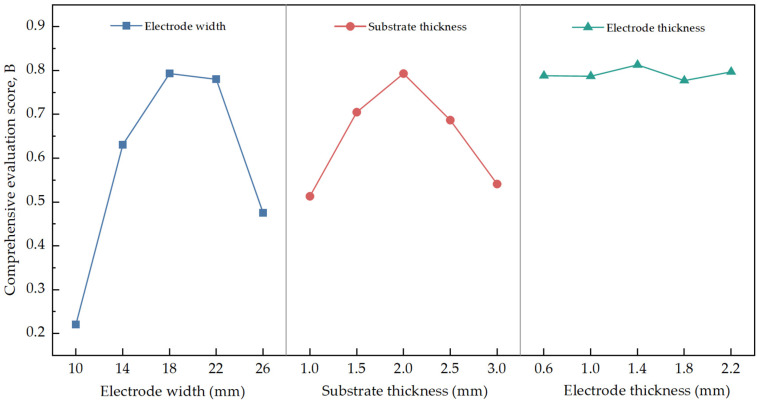
Normalized comprehensive scores of different structural parameters.

Capacitance sensitivity and sensitivity-field uniformity represent two complementary aspects of sensor performance. The former reflects the magnitude of the capacitance response to dielectric variation, whereas the latter reflects the spatial balance of the sensing field within the measured cross-section. An excessive emphasis on capacitance sensitivity may result in a highly concentrated sensitivity field, while an excessive emphasis on uniformity may weaken the overall capacitance response. Because no established theoretical or application-specific basis was available for prioritizing either indicator, equal weights were adopted as a neutral weighting scheme in the primary evaluation. The comprehensive evaluation score B was calculated as follows:

(17) $$B=w_{s}S_{C}^{*}+w_{u}D_{S}^{*}$$

where $w_{s}$ and $w_{u}$ are the weights assigned to the normalized capacitance sensitivity and sensitivity-field uniformity, respectively, and $w_{s}+w_{u}=1$. In the primary evaluation, $w_{s}=w_{u}=0.5$. A larger B indicates a better balance between capacitance sensitivity and sensitivity-field uniformity. The normalized comprehensive scores for the three structural parameters are shown in Figure 8.

As shown in Figure 8, the comprehensive score varied with different structural parameters. For electrode width, the score increased first and then decreased, with the highest value at 18 mm. Although a wider electrode enhanced capacitance sensitivity, excessive electrode coverage increased sensitivity-field non-uniformity. For substrate thickness, the maximum score was obtained at 2.0 mm, indicating a suitable balance between electric-field coupling and field uniformity. In contrast, electrode thickness had a limited effect on the comprehensive score, and the highest value appeared at 1.4 mm.

Based on the normalized comprehensive evaluation, the optimized sensor configuration was determined as an electrode width of 18 mm, a substrate thickness of 2.0 mm, and an electrode thickness of 1.4 mm. This configuration was used for the subsequent capacitance measurements.

### 3.2. Vertical Air-Void Distribution Characterization

#### 3.2.1. Effect of Mixture Gradation on Vertical Air-Void Distribution

**Figure 9:**
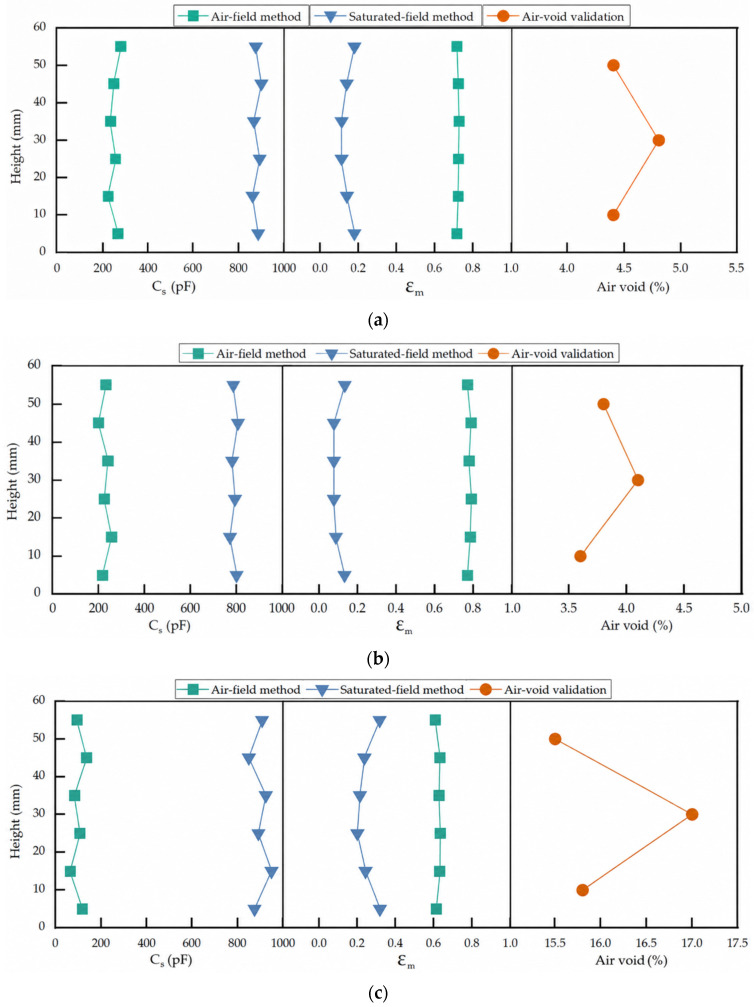
Vertical air-void characterization of Marshall-compacted mixtures with different gradations: (**a**) AC-13; (**b**) SMA-13; (**c**) PAC-13.

The vertical air-void distributions of the Marshall-compacted AC-13, SMA-13, and PAC-13 specimens were evaluated using the capacitance sum, $C_{s}$; the mean meso-scale dielectric constant, $\varepsilon_{m}$; and layer-wise air-void validation. The first two mixtures were compacted dense or skeleton-dense mixtures, whereas PAC-13 represented an open-graded mixture with a higher connected air-void content. The results are shown in Figure 9. Capacitance measurements were conducted at six discrete cross-sections located at 5, 15, 25, 35, 45, and 55 mm above the specimen bottom. After capacitance testing, the representative specimens were divided into lower, middle, and upper layers for air-void validation.

Accordingly, the agreement discussed below refers to the vertical distribution pattern represented by the capacitance indicators and the three validation layers. For the capacitance sum, $C_{s}$, the saturated-field method produced much larger responses than the air-field method. This difference was mainly caused by the high permittivity of water filled in the connected voids. The variation in $C_{s}$ along the specimen height also showed similar characteristics to the layer-wise air-void validation results. Sections with a higher air-void content generally corresponded to stronger capacitance responses, indicating that $C_{s}$ can reflect the overall dielectric change caused by vertical air-void variation.

The mean meso-scale dielectric constant, $\varepsilon_{m}$, provided a clearer indicator for evaluating the accuracy of the reconstructed dielectric field. Under the air-field method, $\varepsilon_{m}$ varied only slightly along the height direction. The differences among the top, middle, and bottom sections were not obvious, indicating that the air-field method had limited sensitivity to the internal void structure. In contrast, the saturated-field method showed more pronounced fluctuations in $\varepsilon_{m}$. This enhanced variation arises because water entering accessible connected voids introduces a much greater dielectric contrast than that provided by air, thereby amplifying the capacitance response of locally void-rich regions. This result indicates that the saturated field improved the dielectric contrast between void-rich and void-poor regions, thereby enhancing the ability to identify vertical heterogeneity.

For the three Marshall-compacted specimens, the $\varepsilon_{m}$ curves obtained from the saturated-field method generally showed a basin-shaped distribution, with higher values near the top and bottom and lower values near the middle. This trend was most evident for PAC-13. This result suggests that the middle region of the Marshall specimen was more compacted, while the upper and lower regions retained relatively higher void contents. During Marshall compaction, repeated impact loading produces non-uniform stress transfer and aggregate rearrangement along the specimen height. The internal region tends to form a relatively stable aggregate skeleton, whereas the upper and lower regions are more strongly affected by loading-surface disturbance, rebound, mold friction, and boundary effects. This vertical pattern is consistent with the layer-wise air-void validation results.

#### 3.2.2. Effect of Air-Void Content on Vertical Distribution in PAC-20 Mixtures

**Figure 10:**
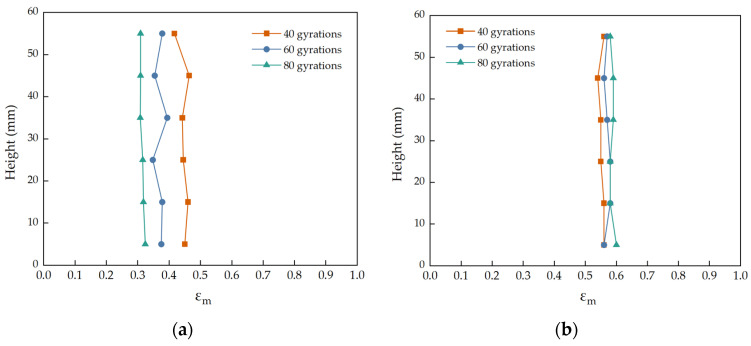
Vertical variation in $\varepsilon_{m}$ for gyratory-compacted PAC-20 specimens with different air-void contents: (**a**) saturated-field method; (**b**) air-field method.

The vertical dielectric response of gyratory-compacted PAC-20 specimens was further analyzed. The following three compaction levels were considered: 40, 60, and 80 gyrations; the corresponding air-void contents, measured by the volumetric method, were 25.2%, 21.4%, and 19.9%, respectively. The dielectric response was measured at the same six cross-sections located 5–55 mm above the specimen bottom. Each plotted point in Figure 10 represents the $\varepsilon_{m}$ value obtained at one discrete cross-section, and the connecting lines are included only to indicate the vertical variation.

As shown in Figure 10, the six discrete $\varepsilon_{m}$ measurements formed relatively stable vertical profiles under both measurement methods. This indicates that gyratory compaction produced a comparatively uniform internal structure. The combined action of axial pressure and shear deformation promotes continuous aggregate rotation, translation, and rearrangement, thereby reducing unstable voids and local structural differences along the specimen height. During gyratory compaction, the mixture is subjected to both vertical pressure and shear action, which promotes aggregate rearrangement and the formation of a stable skeleton. As a result, the air-void distribution becomes more uniform along the specimen height.

Under the air-field method, the $\varepsilon_{m}$ curves showed only slight variations at different heights. The differences among the top, middle, and bottom sections were small, suggesting that the air-field method had limited sensitivity to small changes in the void structure. Under the saturated-field method, the overall profiles were also stable, but local fluctuations were more evident. This is because the saturated field provides a higher dielectric contrast after water fills the connected voids. Therefore, the saturated-field method is more sensitive to local variations in the internal void structure.

With an increasing gyration number, the fluctuation amplitude of $\varepsilon_{m}$ under the saturated-field method gradually decreased. This further confirms that higher gyratory compaction improved the structural uniformity of PAC-20 specimens. The 40-gyration specimen, with the highest air-void content, showed larger local variation, whereas the specimens compacted with higher gyration numbers exhibited smoother vertical profiles.

**Figure 11:**
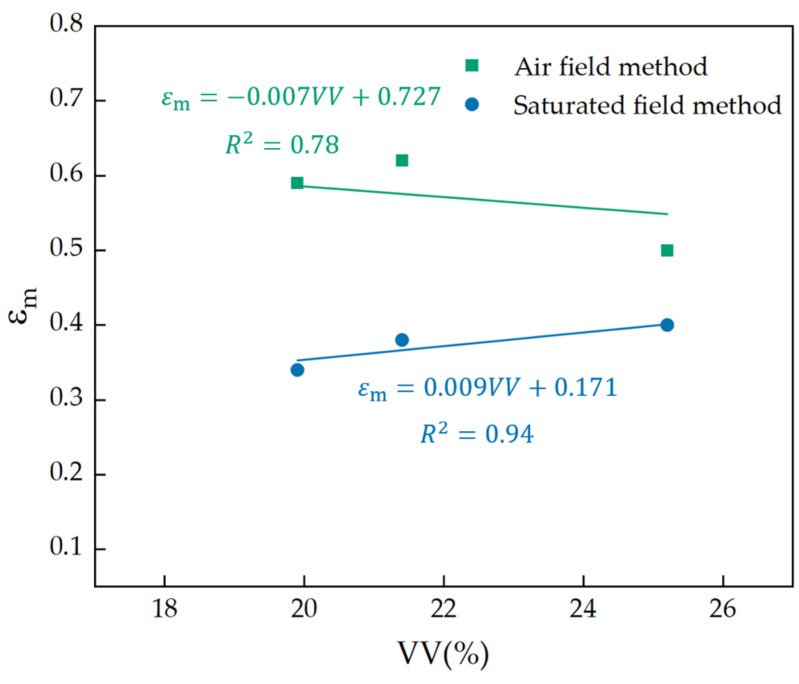
Relationship between average $\varepsilon_{m}$ and measured air-void contents for gyratory-compacted PAC-20 specimens.

To further examine the variation in dielectric response among the three air-void levels, the mean $\varepsilon_{m}$ value averaged over the six measured cross-sections was calculated for each specimen and plotted against the corresponding overall air-void content, as shown in Figure 11. Linear fitting was performed to provide a preliminary quantitative description of the observed variation.

As shown in Figure 11, the mean $\varepsilon_{m}$ obtained using the saturated-field method exhibited an approximately increasing trend with air-void content within the tested range. The linear fitting yielded an R^2^ value of 0.94. This trend can be attributed to the greater volume of water-accessible connected voids at higher air-void contents, which increased the saturated dielectric response. In comparison, the air-field results showed a weaker decreasing trend, with an R^2^ value of 0.78. These results preliminarily indicate that the saturated-field response is more sensitive to changes in the air-void structure. Further tests involving additional specimens and air-void levels are needed to confirm the stability of the observed trends.

### 3.3. Radial Air-Void Distribution Characterization

#### 3.3.1. Radial Distribution Characteristics Under Different Mixture Types and Compaction States

The radial air-void distribution was characterized based on the reconstructed meso-scale dielectric field. The measured cross-section was divided into four radial regions from the center to the edge, denoted as Region 1, Region 2, Region 3, and Region 4. Region 1 represents the central area, while Region 4 represents the region near the specimen edge. The characteristic dielectric constant of each region was extracted to describe the radial variation in air-void distribution.

**Figure 12:**
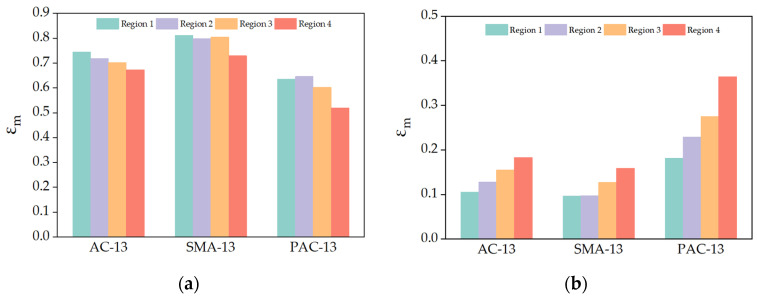
Radial characteristic dielectric constants of Marshall-compacted mixtures with different gradations: (**a**) air-field method; (**b**) saturated-field method.

Figure 12 shows the radial dielectric distributions of Marshall-compacted AC-13, SMA-13, and PAC-13 specimens. Under the air-field method, the characteristic dielectric constants generally decreased from the inner region to the outer region. This trend was more evident for PAC-13. The result indicates that the air-void structure near the mold wall differed from that in the inner region. The outer region was less effectively compacted, while the inner region showed a denser structure.

Under the saturated-field method, the differences among the four radial regions became more pronounced. This indicates that the saturated field enhanced the dielectric contrast related to air-void distribution. For PAC-13, the radial difference was the most significant because of its open-graded structure and higher connected air-void content. Its developed connected pore network allowed for greater regional differences in water filling, thereby amplifying the dielectric contrast between the inner and outer regions. After saturation, water filled the connected voids and amplified the dielectric response in void-rich regions. Therefore, the saturated-field method showed higher sensitivity to radial air-void variation than the air-field method.

**Figure 13:**
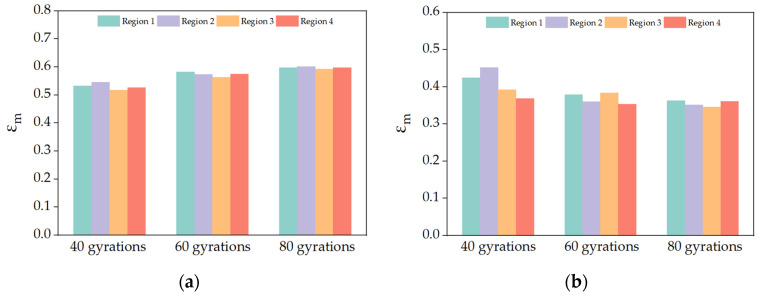
Radial characteristic dielectric constants of gyratory-compacted PAC-20 specimens with different gyration numbers: (**a**) air-field method; (**b**) saturated-field method.

Figure 13 presents the radial dielectric distributions of the gyratory-compacted PAC-20 specimens. Different from the Marshall-compacted specimens, the characteristic dielectric constants of the PAC-20 specimens were more stable among the four radial regions. No clear monotonic increase or decrease was observed under either measurement method. This suggests that gyratory compaction produced a more uniform radial air-void distribution.

The more uniform radial response of the PAC-20 specimens can be attributed to the compaction mechanism. During gyratory compaction, the mixture is subjected to vertical pressure and shear action. This promotes aggregate rearrangement and the formation of a stable skeleton structure. As the gyration number increased, the radial fluctuation of the characteristic dielectric constant decreased, indicating improved structural uniformity.

#### 3.3.2. Quantitative Evaluation of Radial Air-Void Distribution

To further quantify the radial variation in air-void distribution, the edge-to-center ratio, $R_{e/c}$, and radial dielectric gradient, $G_{r}$, were calculated from the characteristic dielectric constants of Region 1 and Region 4. $R_{e/c}$ describes the relative dielectric response of the edge region compared with the central region, while $G_{r}$ reflects the dielectric change rate from the center to the edge. A value of $R_{e/c}$ close to 1 and a $G_{r}$ value close to 0 indicate a more uniform radial distribution.

As shown in Table 3, the Marshall-compacted specimens exhibited more pronounced radial differences than the gyratory-compacted PAC-20 specimens. Under the saturated-field method, the edge regions generally showed stronger dielectric responses than the central regions, especially for PAC-13. This is mainly related to its open-graded structure and higher connected air-void content.

For the gyratory-compacted PAC-20 specimens, the radial indicators remained close to the uniform-state values under both measurement methods. This indicates that gyratory compaction produced a more uniform radial air-void distribution. Overall, the quantitative results confirm that the saturated-field method is more sensitive to radial air-void variation, particularly in mixtures with more developed connected voids.

### 3.4. Repeatability of the Saturated-Field Measurement

To evaluate the repeatability of the saturated-field measurement, the following two tests were conducted: a rotation-angle test and a standing-time stability test. The capacitance sum, $C_{s}$, mean meso-scale dielectric constant, $\varepsilon_{m}$, edge-to-center ratio, $R_{e/c}$, and radial dielectric gradient, $G_{r}$, were used as evaluation indicators. The coefficient of variation, CV, and maximum relative deviation, Max RD, were calculated to quantify measurement stability.

As shown in Table 4, the four indicators changed only slightly with specimen rotation. The CV values of $C_{s}$ and $\varepsilon_{m}$ were 0.60% and 1.03%, respectively, indicating stable overall capacitance and dielectric responses. The CV values of $R_{e/c}$ and $G_{r}$ were slightly higher because these two indicators are more sensitive to local radial differences. However, their variations remained within a limited range. This indicates that the saturated-field measurement was not significantly affected by specimen orientation.

Table 5 shows the stability of the saturated condition under different standing times. The CV values of all indicators were low, and no obvious drift was observed from 3 to 15 min. This suggests that the water-filled reference field remained stable during the measurement period. The small variations in $R_{e/c}$ and $G_{r}$ also indicate that the radial distribution indicators were not strongly affected by short-term water redistribution. Overall, the saturated-field method showed good within-specimen repeatability and short-term stability under controlled conditions. Because the tests used the same specimen, system, and operator, the reported variations mainly reflect specimen positioning and reference-field stabilization. Inter-specimen and inter-operator reproducibility were not separately evaluated and will be investigated using replicate specimens and multiple operators in future studies.

These results also support the potential application of the proposed method in laboratory quality control and the evaluation of field-extracted pavement cores. In laboratory testing, the capacitance-based indicators can be used to compare the vertical and radial air-void distributions of mixtures prepared with different gradations, compaction methods, and compaction levels. For construction quality control and routine pavement evaluation, cylindrical cores extracted from representative pavement locations may be tested to identify depth-dependent compaction non-uniformity and localized void-rich regions that cannot be described by a single bulk air-void value. Because the capacitance measurement does not damage the specimen, the same core can subsequently be used for conventional volumetric, mechanical, or durability tests.

Nevertheless, several limitations should be considered. The saturated-field response is mainly enhanced by water entering accessible connected voids, whereas isolated or poorly connected voids may remain partly air-filled. The current procedure also requires specimen saturation, cylindrical specimen geometry, controlled positioning within the acrylic cylinder, and a consistent annular filling condition. Therefore, the present annular configuration is more suitable for laboratory-compacted specimens and field-extracted cores than for direct in situ pavement measurement. Further development of portable sensor configurations and automated capacitance acquisition would be required for more efficient field implementation.

## 4. Conclusions

1. A movable annular capacitive sensor was developed for air-void distribution characterization of asphalt mixtures. Based on finite-element analysis, the optimized sensor configuration was determined as an 18 mm electrode width, 2.0 mm substrate thickness, and 1.4 mm electrode thickness.

2. The saturated-field method provided stronger dielectric contrast than the air-field method. For the Marshall-compacted AC-13, SMA-13, and PAC-13 specimens, the saturated-field dielectric response better reflected the vertical air-void variation and showed good agreement with the layer-wise air-void validation.

3. For the gyratory-compacted PAC-20 specimens, the dielectric response became more stable as the gyration number increased, indicating an improved internal structural uniformity with higher compaction levels.

4. Radial characterization showed that the Marshall-compacted specimens, especially PAC-13, had more evident center-to-edge variation, whereas the gyratory-compacted PAC-20 specimens exhibited a more uniform radial distribution.

5. Repeatability tests under different rotation angles and standing times confirmed that the saturated-field measurement had good stability, supporting its use as a rapid and non-destructive method for characterizing air-void distribution in asphalt mixtures.

## Figures and Tables

**Table 1:** Basic properties of the asphalt binders.

Performance	SBS-Modified Asphalt Binder	High-Viscosity Asphalt Binder
Penetration (25 °C, 100 g, 5 s, 0.1 mm)	84	53
Softening point (°C)	83	78
Ductility (5 cm/min, 5 °C, cm)	42	28
Viscosity (135 °C, Pa·s)	2.0	2.2

**Table 2:** Specimen information.

Mixture Type	Compaction Method	Compaction Level	Measured Height/mm
AC-13	Marshall compaction	Standard	63.6 ± 0.4
SMA-13	Marshall compaction	Standard	63.4 ± 0.5
PAC-13	Marshall compaction	Standard	63.8 ± 0.6
PAC-20	Gyratory compaction	40 gyrations	66.5 ± 0.7
PAC-20	Gyratory compaction	60 gyrations	64.2 ± 0.5
PAC-20	Gyratory compaction	80 gyrations	62.3 ± 0.5

**Table 3:** Quantitative indicators of radial air-void distribution.

Specimen	Compaction Method	Measurement Method	$\varepsilon_{r,1}$	$\varepsilon_{r,4}$	$R_{e/c}$	$G_{r}$ (mm^−1^)
AC-13	Marshall	Air field	0.74	0.67	0.91	−0.0020
AC-13	Marshall	Saturated field	0.105	0.184	1.75	0.0023
SMA-13	Marshall	Air field	0.81	0.73	0.90	−0.0023
SMA-13	Marshall	Saturated field	0.097	0.159	1.64	0.0018
PAC-13	Marshall	Air field	0.63	0.52	0.83	−0.0031
PAC-13	Marshall	Saturated field	0.182	0.365	2.01	0.0052
PAC-20-40	Gyratory	Air field	0.53	0.52	0.98	−0.0003
PAC-20-40	Gyratory	Saturated field	0.42	0.37	0.88	−0.0014
PAC-20-60	Gyratory	Air field	0.58	0.57	0.98	−0.0003
PAC-20-60	Gyratory	Saturated field	0.38	0.35	0.92	−0.0009
PAC-20-80	Gyratory	Air field	0.59	0.60	1.02	0.0003
PAC-20-80	Gyratory	Saturated field	0.36	0.36	1.00	0.0000

**Table 4:** Repeatability results under different rotation angles.

Rotation Angle	$C_{s}$	$\varepsilon_{m}$	$R_{e/c}$	$G_{r}$ (mm^−1^)
0°	912 (pF)	0.368	1.96	0.0050
90°	905 (pF)	0.362	1.91	0.0048
180°	918 (pF)	0.371	1.98	0.0051
270°	909 (pF)	0.366	1.94	0.0049
Mean	911 (pF)	0.367	1.95	0.0050
CV/%	0.60	1.03	1.53	2.61
Max RD/%	0.77	1.30	1.93	3.03

**Table 5:** Stability results under different standing times.

Standing Time	$C_{s}$	$\varepsilon_{m}$	$R_{e/c}$	$G_{r}$ (mm^−1^)
3 min	908 (pF)	0.365	1.94	0.0049
5 min	913 (pF)	0.369	1.97	0.0050
10 min	910 (pF)	0.367	1.95	0.0049
15 min	906 (pF)	0.364	1.93	0.0048
Mean	909.25 (pF)	0.366	1.95	0.0049
CV/%	0.33	0.61	0.88	1.67
Max RD/%	0.41	0.75	1.16	2.04

## Data Availability

Data are contained within the article.
